# Reconfigurable Liquid Whispering Gallery Mode Microlasers

**DOI:** 10.1038/srep27200

**Published:** 2016-06-03

**Authors:** Shancheng Yang, Van Duong Ta, Yue Wang, Rui Chen, Tingchao He, Hilmi Volkan Demir, Handong Sun

**Affiliations:** 1 Division of Physics and Applied Physics, School of Physical and Mathematical Sciences, Nanyang Technological University, Nanyang Link, 637371, Singapore; 2 Department of Physics, King’s College London, Strand, London, WC2R 2LS, UK; 3 Department of Electrical and Electronic Engineering, South University of Science and Technology of China, Shenzhen, Guangdong, 518055, P. R. China; 4 College of Physics Science & Technology, Shenzhen University, Shenzhen, Guangdong, 518060, P. R. China; 5 Centre for Disruptive Photonic Technologies (CDPT), Nanyang Technological University, Nanyang Link, 637371, Singapore; 6 School of Electrical and Electronic Engineering, Luminous! Center of Excellence for Semiconductor Lighting and Displays, Nanyang Technological University, Nanyang Avenue, 639798, Singapore; 7 Department of Electrical and Electronics Engineering, Department of Physics and UNAM-National Nanotechnology Research Center, Bilkent University, Bilkent, 06800 Ankara, Turkey

## Abstract

Engineering photonic devices from liquid has been emerging as a fascinating research avenue. Reconfigurably tuning liquid optical micro-devices are highly desirable but remain extremely challenging because of the fluidic nature. In this article we demonstrate an all-liquid tunable whispering gallery mode microlaser floating on a liquid surface fabricated by using inkjet print technique. We show that the cavity resonance of such liquid lasers could be reconfigurably manipulated by surface tension alteration originated from the tiny concentration change of the surfactant in the supporting liquid. As such, remarkable sensing of water-soluble organic compounds with a sensitivity of free spectral range as high as 19.85 THz / (mol · mL^−1^) and the detectivity limit around 5.56 × 10^−3^ mol · mL^−1^ is achieved. Our work provides not only a novel approach to effectively tuning a laser resonator but also new insight into potential applications in biological, chemical and environmental sensing.

Optical microresonators confine light into tiny volumes where the light‒matter interactions are strongly enhanced. In addition to the fundamental physics interest for the investigation of cavity quantum electrodynamics, microresonators can find numerous practical applications including sensitive sensors^1^, optical filters^2^ and switches^3^, quantum information processing^4^ and novel light sources^5^. Various types of microcavities, such as Fabry-Perot cavities, whispering gallery mode (WGM) cavities, photonic crystal and random cavities have been demonstrated^6^,^7^,^8^,^9^,^10^. Among all the configurations, WGM microresonators are especially interesting^11^. Firstly, with appropriate optical gain, the high-quality (*Q*) factor of WGM resonator should ensure low threshold lasing upon proper pumping^12^. Secondly, in a WGM microresonator, an evanescent field exists tens to hundreds of nanometers near the cavity’s interface. This evanescent field can interact with the surrounding environment and dramatically change the resonator characteristics such as resonant wavelength, intensity and *Q*-factor, which allows for extremely sensitive detection down to even a single molecule level^13^,^14^,^15^,^16^. The evanescent field inherent with a WGM resonator can be further exploited to couple among waveguides and resonators, leading to important devices such as optical filter and add-drop routers for optical communication^2^. More interestingly, a WGM resonator is actually a ring laser cavity which exhibits two whispering gallery modes degenerate in frequency corresponds to clockwise and counterclockwise travelling waves. Bistability between these two counter-propagating travelling waves has been demonstrated for semiconductor ring lasers and it is the corner stone on which all-optical memories and logic gates devices have been proposed^17^,^18^. Very recently, scientists have constructed coupled WGM cavity systems combining balanced loss and gain, which provides a unique platform to implement classical analogues of quantum systems described by non-Hermitian parity–time (PT)-symmetric Hamiltonians. By exploiting PT symmetry made of two coupled active-passive-WGM microresonators for device applications, the first resonant on-chip optical device exhibiting PT-symmetry breaking has been demonstrated and it can act as an efficient nonlinear optical isolator^19^,^20^. Up to now, most WGM based microcavities or microlasers are made from solid state materials with engineered geometries. On the other hand, liquid has been paid increasing attention for constructing photonic devices such as passive light manipulating device^21^,^22^ and microlasers^23^,^24^,^25^,^26^, given that liquid devices are compatible to biological environment.

In view of practical applications, microcavities are desirable to be flexible, reversible and tunable in wavelength. Conventional semiconductor-based ones fabricated by top-down and bottom-up approaches^2^,^10^,^27^,^28^ failed to achieve these characteristics due to their rigid nature, which limits the practical value of WGM microcavities. Comparatively, polymer microresonators have the nature of elasticity, flexible doping, and simple fabrication^1^,^6^,^29^, which enables them to be ideal candidates for tunable microlasers. Up to now, plentiful ways have been employed to tune the lasing spectra by tailoring the refractive index and the cavity size of polymer microcavities. By slightly deforming the dye-doped microdroplets in polymer matrix, we observed shift of WGM lasing^30^ and demonstrated the tuning from polymer fibers via changing their effective refractive index through strain^31^. M. Humar, *et al.* applied external electrical fields on a liquid crystal microcavity to achieve wavelength tuning^32^. A. Kiraz, *et al.* manipulated the resonant peaks by changing the environmental humidity of microdroplets on superhydrophobic surface^33^ and Sindy K. Y. Tang, *et al.* dissolved the microdroplets made by Benzyl alcohol into the water carrier to continuously decrease the cavity sizes to achieve wavelength shift^34^. However, all the previous works for the lasing spectra tuning suffer from various problems, such as small tunable range^31^, short working lifetime^34^, complex setup in order to provide external electric or magnetic field^32^,^35^ and non-bidirectional tuning^34^. In particular, it remains challenging to reconfigurably tune a liquid droplet resonator. Herein, we demonstrate a new type of liquid droplet microlasers floating on water surface. The resonator shape as well as size of the quasi-disk WGM lasers can be changed by the variation of surface tension of water through employing surfactant, which provide reconfigurable tuning of resonance and output envelop. The maximum tuning range of output envelop can reach ~10 nm. Furthermore, the sensing application for water-soluble organic compounds exemplified by ethanol is proposed and demonstrated with the FSR sensitivity as high as 19.85 THz/(mol · mL^−1^) and detectivity limit around 5.56 × 10^−3^ mol · mL^−1^.

## Results

### Quasi-disk liquid microlaser floating on water surface

**Figure 1:**
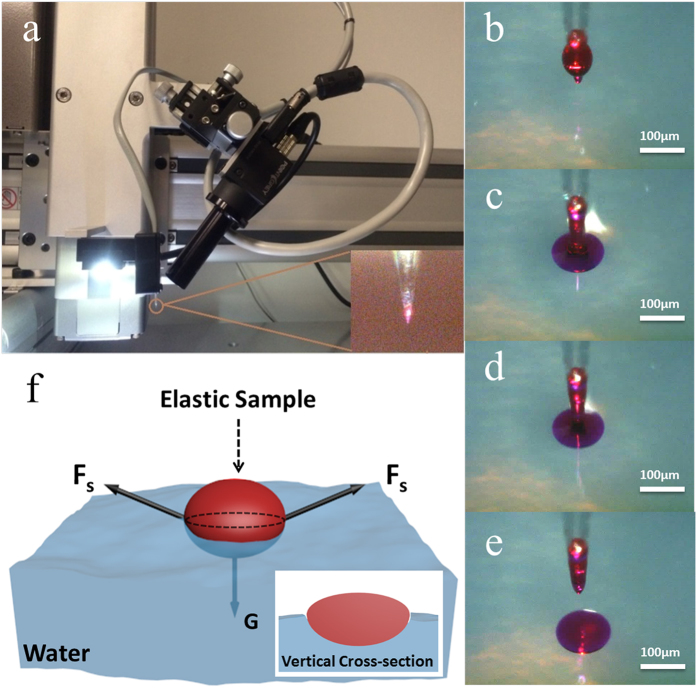
The fabrication and force analysis of the floating microlaser. (**a**) The overview picture of microplotter. The inset shows the hollow dispenser with dye-doped epoxy resin solution. (**b**–**e**) The fabrication steps of a floating microlaser. (**b**) Approaching the dispenser to soap water. (**c**) Touching the dispenser with soap water surface. (**d**) Lifting up the dispenser. (**e**) A self-assembled floating microlaser. (**f**) The force analysis of the floating microlaser. The inset shows the vertical cross-section of the microlaser. The asymmetric ellipsoid shape is attributed to the different surface tensions at the interfaces of droplet/water and droplet/air.

In preparation of the microlasers, Rhodamine 6 G (R6G), dichloromethane and epoxy resin were mixed together with proper ratio and the liquid droplet microlasers were carefully fabricated on the surface of water solution by using a microplotter (Fig. 1a–e and *Methods*), which is superior in the fabrication of soft-matter microcavities due to the precious control of the diameter and position^36^,^37^,^38^,^39^. Meanwhile, soap water was exploited to manipulate the surface tension of water. The force analysis of the floating microdroplet is shown in Fig. 1f. Instead of immersing into water, the liquid droplet floated on the top of water surface like a water strider (Fig. 1f inset). The surface tension forces of water around the cavity are in equilibrium with the gravity force. The precise shape of the droplet depends on the surface tensions at both interfaces of droplet/water and droplet/air. However, the horizontal cross-section should be circular to ensure the minimum free energy. Thus a WGM resonator is naturally formed and the dash line marked plane is the fundamental optical plane of WGM lasing.

**Figure 2:**
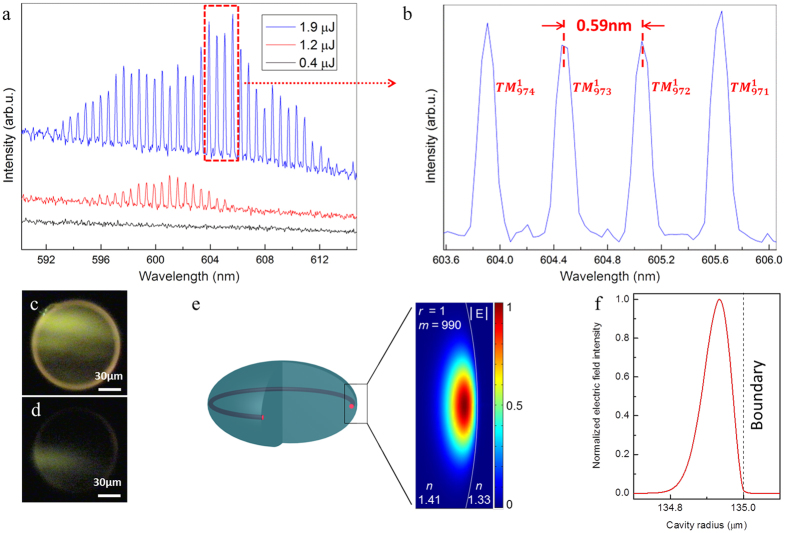
The lasing performances of the floating microlaser. (**a**) The PL emission of a single floating microlaser. (**b**) Enlarged lasing spectrum. (**c**) Optical image of the stimulated emission. (**d**) Optical image of the spontaneous emission. (**e**) Schematic of the floating microlaser and the electric energy distribution of a fundamental WGM with a mode number of 990. (**f**) Normalized electric field intensity distribution of the first order WGM in radial direction. The dashed line illustrates the boundary of the cavity.

Figure 2a shows the photoluminescence (PL) spectrum from a quasi-disk microlaser floating on soap water. It can be seen that under low excitation intensity, only the spontaneous emission was observed. When the excitation energy was around 1.2 μJ, the spectrum collapsed from broadband spontaneous emission to sharp lasing peaks. At 1.9 μJ excitation power, the intensity of the lasing peaks was dramatically increased and more modes emerged, which further supported the lasing phenomenon. The bright ring above the threshold (Fig. 2c) suggests the lasing mechanism to be whispering gallery mode, which is further confirmed by analyzing the lasing spectra. It is known that the WGM lasing can be characterized by three parameters: mode number *m*, radial mode number *r*, and azimuthal peak number *l* − *m* + *1*^40^. The fundamental mode (*r* = *1, m* = *l*) is reasonable to be applied here because the microlaser is a thin quasi-disk floating on soap water surface. Thus, the lasing mechanism can be analyzed by applying a 2D WGM model and the free spectral range (FSR) can be calculated from the following equation^5^,


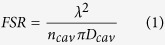


where *λ* is the resonant wavelength, 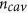 and 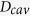 are the refractive index and the diameter of the cavity, respectively. The calculated 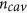 can be found from our previous work^5^ and 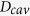 is estimated from the PL images. By substituting *n*_*cav*_ = 1.41 and *D*_*cav*_ = 135 μm, the theoretical FSR is 0.61 nm, which is very close to 0.59 nm of the experimental observation (Fig. 2b). It is also known that the resonant wavelengths of WGM cavities are relevant to the cavity size *D*_*cav*_, refractive index contrast of the cavity material 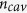 and the surrounding medium 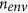, radial mode number *r* and the longitudinal mode number *m* with the relation of^11^,^31^


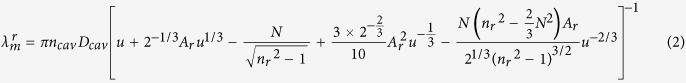


where


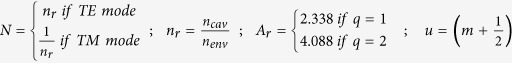
.

After calculating with *n*_*env*_ = 1.33, the lasing peaks fit well with the fundamental WGM transverse magnetic (TM) modes (Fig. 2b). Therefore, the lasing mechanism is revealed to be WGM lasing.

In order to have a better understanding of the WGM lasing inside the floating microlaser, numerical simulations were carried out by employing the finite element method (via COMSOL Multiphysics)^41^. Figure 2e displays the electric field distribution of a fundamental WGM in the equator plane of a floating quasi-disk microlaser with a diameter of 135 μm. Light confinement via total internal reflection can be seen clearly at the disk-water interface. The resonance wavelength was set at 604.5 nm, which is exactly the same as the experimental data in Fig. 2a. Thus, the results reveal the real picture of the WGMs inside the floating microlaser. From the electric field intensity distribution plotted in Fig. 2f we can conclude that the field located outside the cavity is only a small part of the profile, which is known as evanescent wave. Further calculation shows that up to 99% energy is confined within the cavity, which lays the foundation of the high quality WGM lasing from the floating microlaser.

### Reconfigurable tuning of cavity by changing surface tension

**Figure 3:**
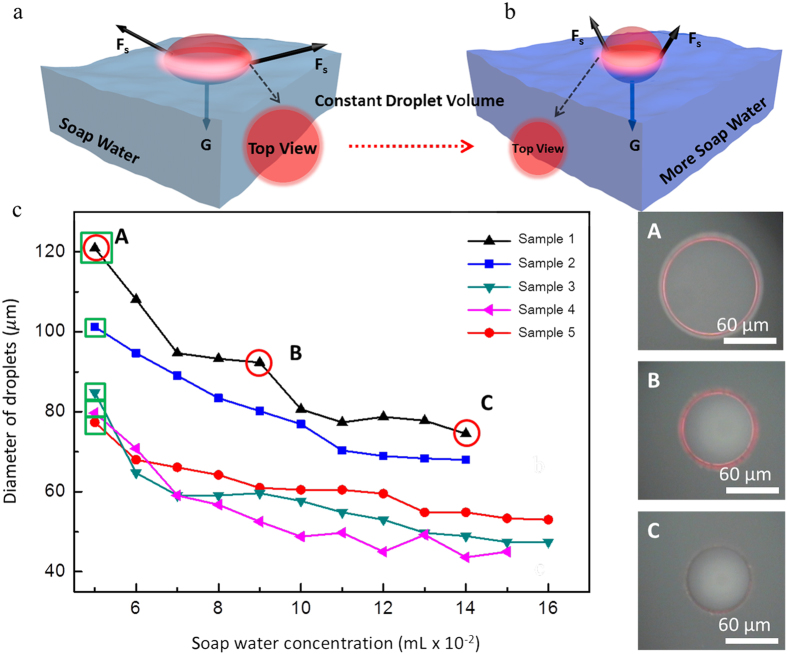
The size changing performance of the floating microlaser. (**a**,**b**) The force analysis of a floating microdroplet when the surface tension of water is modified. It can be seen that the diameter of the WGM plane is reduced from the top view while the total volume of the microdroplet remains the same. (**c**) Experimental results of the size changing performance of floating microlasers with different initial diameters. The highlighted points in rectangle indicate the initial size for each sample. A, B and C represent the optical images of the corresponding points in the line chart.

It is worth noting that the forces from the surface tension of water and the gravitational force should be in equilibrium as the microlaser is floating on water (Fig. 1f). However, once the surface tension at droplet/water interface changes, the depth that the droplet dips on the water surface will change. Because of the deformability nature of polymer, the curvature of the vertical cross-section of the droplet will change accordingly. The surfactant, which is the main component of the liquid soap, can attract the water molecules because of polarity^42^,^43^. Therefore, the attraction between the water molecules is reduced due to the presence of surfactant, which leads to the decrease of the surface tension^42^,^43^. The reduction of surface tension at droplet/water interface results in the increased curvature of the vertical cross-section of the droplet and simultaneously the reduced diameter in the horizontal cross-section, given the constant volume of the droplet (Fig. 3a,b). Figure 3c displays the size changing performance of the microlasers as proposed. The initial state (highlighted in rectangle) for the all the tested samples was 0.6 mL of water mixed with 0.05 mL of soap water and an example of the size changing process is illustrated in the optical images in Fig. 3c A–C. With the increase of the soap water concentration, the size change of the microlasers could reach 40% in average.

**Figure 4:**
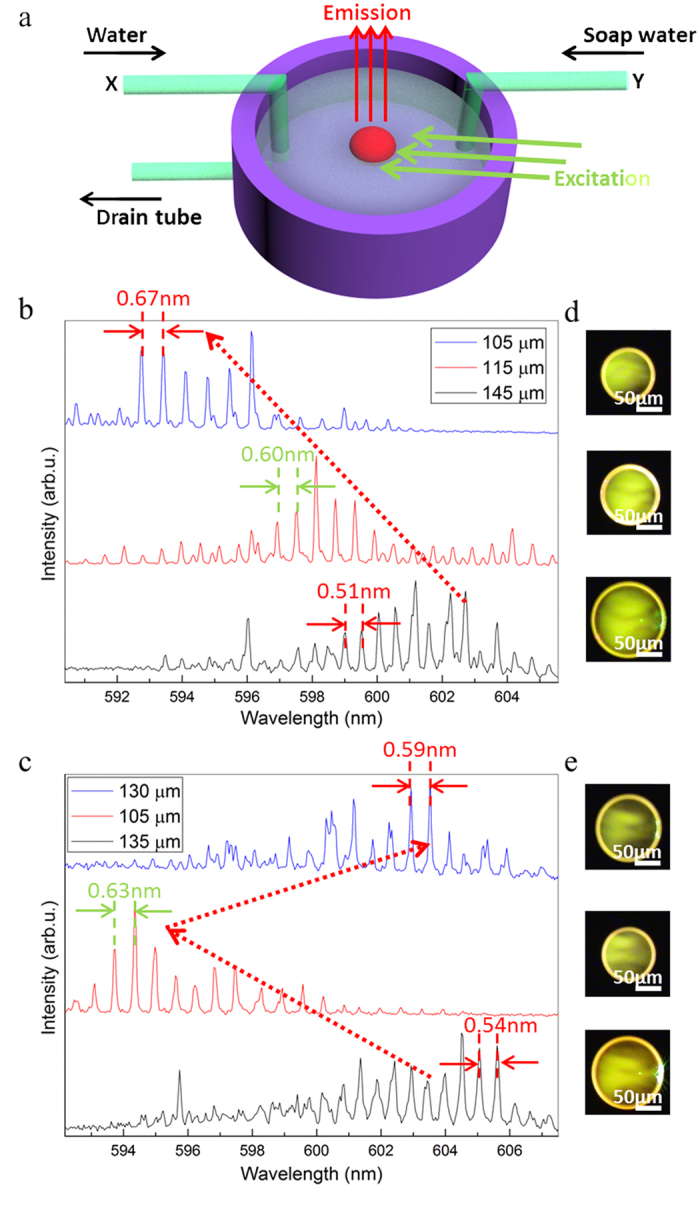
Reconfigurable and bidirectional wavelength tuning of the floating microlaser. (**a**) Illustration of the home-made fluidic system. (**b**) The tuning process of a tunable microlaser floating on soap water. With the diameter decreased from 145 μm to 105 μm, the lasing envelop blueshifted 10 nm. (**c**) Reconfigurable tuning process of a tunable microlaser floating on soap water. The lasing envelop blueshifted 10 nm first and then redshifted 9 nm with the diameter of the microlaser changed from 135 μm to 105 μm to 130 μm. (**d**,**e**) PL images of the corresponding floating microlasers under excitation.

The change in the radius of the horizontal cross section shall induce the shift of the lasing modes, which forms the foundation of the laser tuning. In order to test the wavelength drift, a home-made fluidic system was fabricated. The capillary tube X and Y shown in Fig. 4a were used to control the soap water concentration while the drain capillary tube could keep the liquid volume in the container nearly unchanged, which is important to maintain the position of the excitation spot. As a result, the wavelengths of the emission from the microlaser shifted from ~603 nm to ~593 nm (Fig. 4b), which is relatively large comparing with the previously reported works (~5 nm)^31^,^33^. As the size decreased from 145 μm to 105 μm, which can be seen from the PL images in Fig. 4d, the FSR fit well with the corresponding cavity size so that the lasing mechanism remained unchanged to be WGM lasing.

Normally, big cavities have larger *Q* factor than small ones^44^. However, the situation is reversed in our work. The *Q* factor (defined as *Q* = resonant wavelength/full width at half maximum (FWHM) of the peak)^10^ for the shown 145 μm, 115 μm and 105 μm microlasers were 4000, 5300 and 5800, respectively. Thickness variation of the microlasers can be used to explain. As the total cavity volume did not change during the tuning process, the thickness of the cavity was increased. For thin cavities with large horizontal cross-section, the overlap of the cavity and the mode profile is less than the small ones. Thus, the optical leakage is increased and the *Q* factor is decreased.

The wavelength tuning can be explained from the aspect of output power^34^. It is known that the output power from a microlaser is proportional to the out-coupling efficiency, the gain profile and the mode volume^34^,^45^,^46^. The out-coupling efficiency and mode volume both blueshift with the decreasing of microcavity diameter while the gain profile is unchanged. So as a product of the out-coupling efficiency, the gain profile and the mode volume, the output wavelength will shift to shorter wavelength with the decrease of cavity size.

The reversibility of the floating microlasers is shown in Fig. 4c. We increased the soap concentration first and then diluted the soap water. It can be seen from the PL images in Fig. 4e that the cavity size decreased from 135 μm to 105 μm first, and then reversed to 130 μm after dilution. The lasing spectrum also showed a sensitive response. After blueshifting around 10 nm, the modes redshifted 9 nm, nearly back to the original lasing position. Instead of dissolving or evaporation, our floating microlaser remained a long lifetime under ambient conditions (Supplementary information Fig. 1). The reconfigurable and bidirectional tuning and long lifetime ensure our floating microlaser to be a potential choice for fluidic sensing.

### Sensing

**Figure 5:**
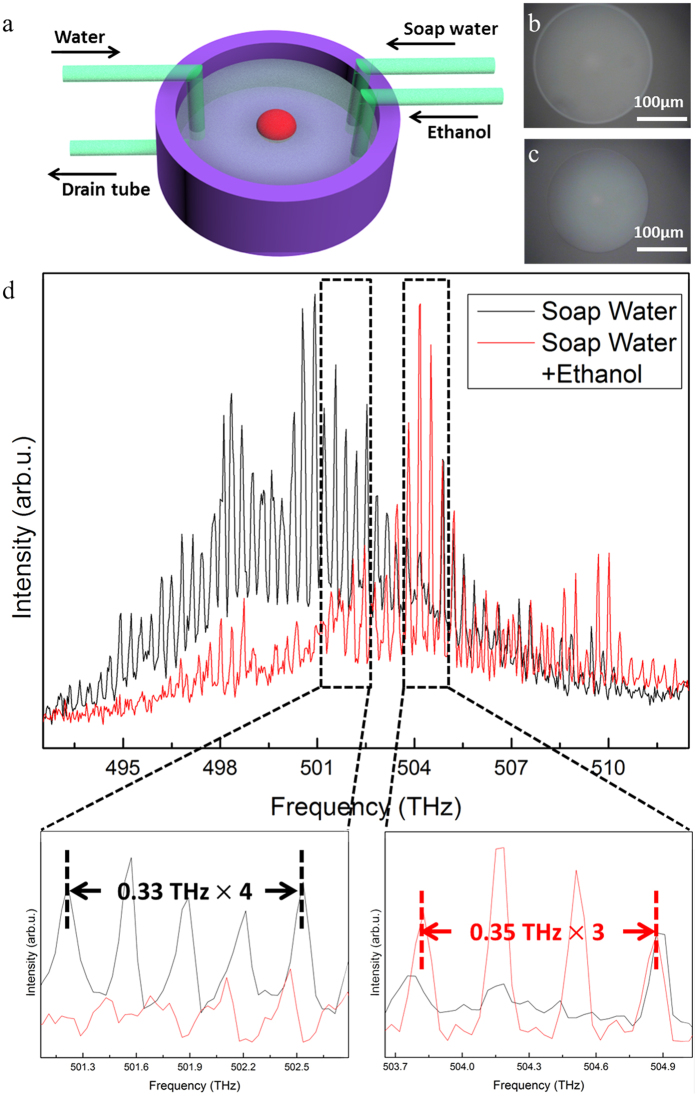
Ethanol sensing via the floating microlaser. (**a**) Modified home-made fluidic system for ethanol sensing. (**b**) PL image of the floating microlaser before adding ethanol. (**c**) PL image of the floating microlaser after adding ethanol. (**d**) The experimental results of ethanol sensing with the floating microlaser.

To further explore the application of our floating microlasers, we demonstrate herein a water-soluble molecules sensing mechanism. As indicated in Fig. 5a, we fabricated another capillary tube in the home-made fluidic system to transport the detected liquid. Ethanol was chosen because it does not react with epoxy resin and dye molecules. More importantly, the ethanol is water-soluble and can attract water molecules to modify the surface tension of water. The initial state of the system was 0.6 mL of water mixed with 0.04 mL of soap water. The ethanol concentration raised from 0–1.007 × 10^−3^ mol · mL^−1^ after adding 0.04 mL of ethanol. Meanwhile, the surface tension of the water was reduced due to the molecular attraction between the polar ethanol and water molecules and therefore, resulted in the diameter variation of the floating microlaser. As shown in Fig. 5b–d, the cavity size decreased from 205–180 μm with the lasing envelop blueshifted around 4 THz. Detailed analysis of the spectrum in the enlarged part of Fig. 5d indicates that the FSR varied from 0.33 THz to 0.35 THz, which leads to a FSR sensitivity as high as 19.85 THz/(mol · mL^−1^). The limit of detection of the sensor is related to the *Q* factor of the cavity, or the FWHM of the resonances. In general, the minimum distinguishable mode shift is the FWHM of the lasing peaks, which is 0.11 THz in our work. Thus, the limit of detection is estimated to be 5.56 × 10^−3 ^mol · mL^−1^. It is reasonable to derive that by increasing the soap water concentration before sensing, which provides smaller floating microlasers with higher *Q* factor in the initial state, the limit of detection can be further improved. This sensing mechanism can also be applied for other water-soluble organic chemicals that can easily manipulate the surface tension of water. Comparing with previously reported WGM sensors^1^,^6^,^11^,^34^, our floating microlaser provides not only competitive sensitivity and limit of detection, but also bidirectional and multifunctional sensing, which is promising in fluidic and biomedical applications.

In conclusion, we have demonstrated a new kind of floating quasi-disk WGM microlaser. By simply modifying the surface tension of water to deform the cavity, manipulation of the lasing peaks was achieved. These tunable microlasers were easily fabricated, and could have a reversible size changing around 40%, which led to a spectral envelop drift up to 10 nm. Bidirectional tuning as well as reconfigurable tuning of lasing peaks were realized by controlling the surfactant concentration. Moreover, a demonstration of ethanol sensing with FSR sensitivity as high as 19.85 THz/(mol · mL^−1^) was proposed and realized. As the limit of detection is estimated around 5.56 × 10^−3^ mol · mL^−1^, it is expected that these floating microlasers are highly sensitive to all the water-soluble organic compounds that do not react with the device. In addition, our work also provides an effective method to tune the lasing peaks, which can be widely applied in fluidic sensing and bio-sensing based on micrometer level.

## Methods

### Microlaser Fabrication

R6G (1.0 mg), serving as the gain media, was mixed with dichloromethane (0.1 mL, purity 99.7%) and epoxy resin (Araldite 506, 400 mg, from Sigma-Aldrich). Epoxy was chosen because of its thermal stability, transparency, high viscosity and immiscibility with water, which enable it to be a good cavity host material for this work^5^. The microlasers were fabricated by a GIX^TM^ Microplotter^TM^ II from Sonoplot, INC (Fig. 1a), which is a computer controlled system. It consists of a dispenser, a camera for imaging and an ultrasonic vibrator for cleaning. Due to adhesion, a little solution was attached outside the wall of the dispenser after the glass micropipette immersed in the solution (Fig. 1b). When touching the soap water surface, the solution left outside the wall of the dispenser was “pulled” down to the soap water surface and self-assembled to be a circular floating microlaser by the surface tension of water (Fig. 1c–e). The initial radius of the floating microlaser can be controlled by the dispenser size as well as the immersion depth when the dispenser touches the epoxy solution. The soap water, which was exploited to manipulate the surface tension of water, was prepared by mixing 25 mL of water and 8 μL of Mama Lemon (Lion Corporation, Japan).

### Measurements

The floating microlasers were placed on an X-Y-Z controlled platform and pumped by a 532 nm Q-switched Nd : YAG laser (pulse width : 1 ns, repetition rate : 60 Hz). The laser beam was focused by a spherical lens and guided to pump the sample with an angle of 45° normal to the platform. The signal from the samples was collected by an objective (50X, numerical aperture = 0.42) and delivered to a silicon charge-coupled device (CCD) for spectrum recording. An intensity attenuator was placed along the optical path to control the excitation power.

## Additional Information

**How to cite this article**: Yang, S. *et al.* Reconfigurable Liquid Whispering Gallery Mode Microlasers. *Sci. Rep.* **6**, 27200; doi: 10.1038/srep27200 (2016).

## Supplementary Material

Supplementary Information

Supplementary Information
