# Supplementary material for: Reconfigurable Liquid Whispering Gallery Mode Microlasers
Source: Sci Rep. 2016 Jun 3;6:27200. doi: 10.1038/srep27200 (PMC4891658; doi:10.1038/srep27200)

Reconfigurable Liquid Whispering Gallery Mode Microlasers

Shancheng Yang[1], Van Duong Ta[1,2], Yue Wang[1], Rui Chen[1,3], Tingchao He[1,5], Hilmi Volkan Demir[1,4,6,7], and Handong Sun[1,4,*]

[1]*Division of Physics and Applied Physics, School of Physical and Mathematical Sciences, Nanyang Technological University, Nanyang Link, Singapore, 637371.*

[2]*Department of Physics, King's College London, Strand, London, UK, WC2R 2LS.*

[3]*Department of Electrical and Electronic Engineering, South University of Science and Technology of China, Shenzhen, Guangdong, P. R. China, 518055.*

[4]*Centre for Disruptive Photonic Technologies (CDPT), Nanyang Technological University, Nanyang Link, Singapore, 637371.*

[5]*College of Physics Science & Technology, Shenzhen University, Shenzhen, Guangdong, P. R. China, 518060.*

[6]*School of Electrical and Electronic Engineering, Luminous! Center of Excellence for Semiconductor Lighting and Displays, Nanyang Technological University, Nanyang Avenue, Singapore, 639798.*

[7]*Department of Electrical and Electronics Engineering, Department of Physics and UNAM-National Nanotechnology Research Center, Bilkent University, Bilkent, Ankara, Turkey, 06800.*

[*]Corresponding author(s): Handong Sun, electronic mail: hdsun@ntu.edu.sg

Supplementary Video Legends

Supplementary Video 1: The fabrication of a floating droplet WGM microlaser by inkjet method.

Supplementary Figure Legends

Supplementary Figure 1: Stability of the floating microlaser under ambient conditions. (a) 10 minutes after fabrication. (b) 1 hour after fabrication. (c) 2 hours after fabrication. (d) 4 hours after fabrication. The bright ring at the boundary of the cavity is due to interference and the floating microlaser maintains constant diameter (~100 μm) during the experiment.

Supplementary Figures

Supplementary Figure 1

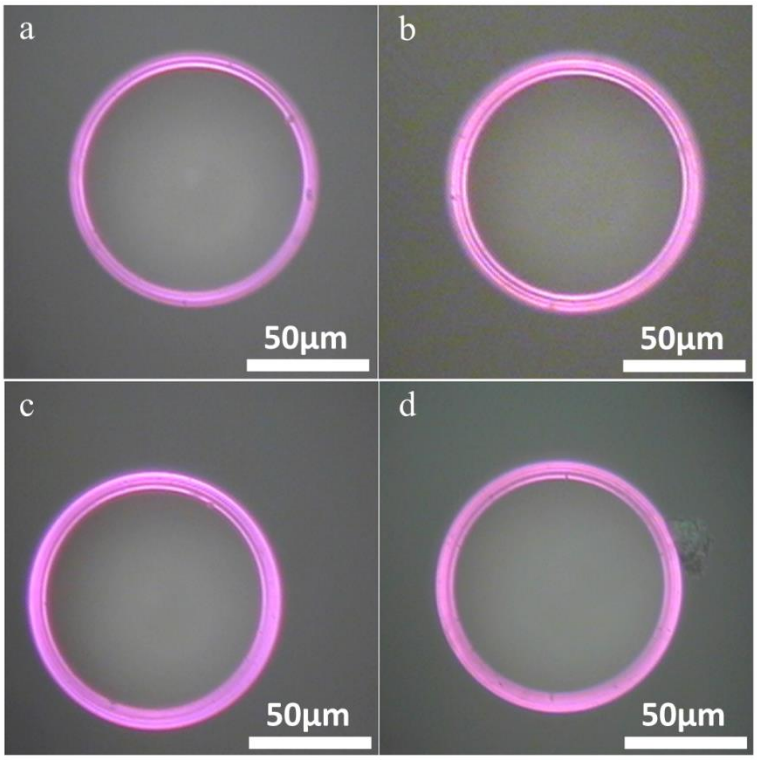

Supplement: Supplementary Information [file srep27200-s2.pdf]
